# Dual-release hydrocortisone treatment improves serum and peripheral blood mononuclear cell inflammatory and immune profiles in patients with autoimmune primary adrenal insufficiency

**DOI:** 10.3389/fimmu.2025.1489254

**Published:** 2025-01-23

**Authors:** Laura Tomasello, Antonina Coppola, Giuseppe Pizzolanti, Carla Giordano, Giorgio Arnaldi, Valentina Guarnotta

**Affiliations:** ^1^ Department of Health Promotion, Mother and Child Care, Internal Medicine and Medical Specialties, Section of Endocrinology, University of Palermo, Palermo, Italy; ^2^ Advanced Technologies Network Center (ATEN Center), University of Palermo, Palermo, Italy

**Keywords:** regulatory T-lymphocytes, interleukin-6, PD-L1, HSP-70, CD4 +/CD8 + ratio, Addison’s disease, flow cytometry

## Abstract

**Objective:**

The primary outcome was the evaluation of the T-cell phenotype in autoimmune primary adrenal insufficiency (PAI). Secondary outcomes included the evaluation of the CD4^+^CD25^+^Foxp3^+^ Treg population and the gene expression levels of IL-6, IL-17A, cyclooxygenase (COX)-2, heat shock proteins (HSP)-70, indoleamine-2,3-dioxygenase (IDO), programmed death-ligand 1 (PD-L1), inducible nitric oxide synthase (iNOS), and thioredoxin (TXN)-1.

**Methods:**

We prospectively included 15 patients with PAI on conventional glucocorticoid (GC) replacement therapy, 15 switched to dual-release hydrocortisone (DR-HC), and 20 healthy controls. Serum inflammatory parameters and peripheral blood mononuclear cells (PBMCs) were evaluated at baseline and after 12 months of treatment.

**Result:**

At baseline, significantly higher CD4^+^ and CD8^+^ (both *p* < 0.001) T-cell percentages, a lower CD4^+^/CD8^+^ ratio (*p* < 0.05), and higher CD25^+^ and CD4^+^/CD25^+^ T cells (both *p* < 0.001) were observed in PAI compared to controls. After 12 months of DR-HC treatment, we found significantly lower IL-6 (*p* = 0.019), IL-17A (*p* = 0.046), COX-2 (*p* < 0.001), HSP-70 (*p* = 0.006), and TXN-1 (*p* = 0.008) and higher PD-L1 (*p* < 0.001) and IDO (*p* < 0.001) mRNA values compared to baseline. After 12 months of DR-HC treatment, a significant increase in CD4^+^ T cells (*p* = 0.012), PD-L1 (*p* = 0.003), and IDO (*p* < 0.001) and a decrease in CD8^+^ T cells (*p* < 0.001), IL-6 (*p* = 0.003), IL-17A (*p* = 0.0014), COX-2 (*p* < 0.001), HSP-70 (*p* = 0.005), and TXN-1 (*p* = 0.0008), as well as a significantly higher conversion in the CD4^+^/CD8^+^ ratio (*p* = 0.033), were observed compared to conventional GCs.

**Conclusions:**

The switch from conventional GCs to DR-HC treatment altered the T lymphocyte phenotype and CD4^+^/CD8^+^ ratio in a Treg-independent manner, inducing significant improvements in the immune and inflammatory profile in PAI.

## 1 Introduction

Primary adrenal insufficiency (PAI) is characterized by inadequate cortisol secretion, due to the destruction of the adrenal gland and a consequent lack of glucocorticoid and mineralocorticoid productions. Autoimmune adrenalitis, or Addison’s disease (AD), is the most common cause of PAI. PAI can occur in isolation or as part of a group of autoimmune disorders known as autoimmune polyendocrine syndrome (APS).

Loss of tolerance to self-antigens in the adrenal cortex progressively leads to AD, primarily driven by self-reactive Th1 cells (1). Autoimmune disorders, including endocrine autoimmune diseases, are often associated with an imbalance between T helper type (Th)-17 and regulatory T lymphocytes (Tregs) (2). Specifically, Treg cells, due to their insufficient numbers or loss of function, fail to limit autoreactivity and establish peripheral self-tolerance (3). However, the role of Tregs in PAI remains a subject of ongoing debate. Recent studies have reported that patients with PAI exhibit similar percentages of Tregs, but with a higher turnover rate that could impair their functionality (2).

Fully functional Treg cells, initially identified as CD4^+^CD25^+^ T cells, are characterized by the expression of the transcription factor Forkhead box P3 (Foxp3). Their surface phenotype is defined by several membrane clusters of differentiation (CDs), with differential expressions closely related to the composition of the extracellular microenvironment (4, 5).

The modulation of proinflammatory cytokines and immunomodulatory factors, notably interleukin (IL)-6 and IL-17A, cyclooxygenase (COX)-2, heat shock protein (HSP)-70, indoleamine-2,3-dioxygenase (IDO), and programmed death-ligand 1 (PD-L1), may differently affect CD4^+^ and CD8^+^ expansion and initiate Treg expansion by different CD4^+^ or CD8^+^ T-cell conversion (2, 4–9). The multiplicity of phenotypes likely reflects the heterogeneity in the efficacy of Treg to counteract CD8^+^ expansion. Furthermore, this variability contributes to the difficulty in elucidating the clinical involvement of Tregs in the progression of pathology (10). Exploring Treg biology and the molecular process involved in their differentiation and activation could provide a valuable opportunity for developing immunotherapy approaches in PAI.

Glucocorticoids (GCs), generally, exert anti-inflammatory and immunosuppressive effects by regulating the expression of genes encoding various inflammatory and immunomodulatory proteins and by modulating the lymphocyte pattern, including Treg induction. However, high doses of glucocorticoids can have immunosuppressive effects, increasing susceptibility to infections and promoting a proinflammatory state (11).

Conventional GC replacement regimens (cortisone acetate and hydrocortisone) remain the cornerstone of treatment for patients with PAI, typically administered in two or three daily doses. However, overexposure to replacement GCs can lead to adverse effects, including an increased risk of infections, diabetes mellitus, and cardiovascular disease, which result in higher mortality rates compared to the general population (12). In recent years, the innovative dual-release hydrocortisone (DR-HC), a novel formulation designed to mimic the physiological circadian rhythm of cortisol secretion, has been associated with improvements in quality of life (QoL), metabolic parameters, and the proinflammatory state in AI patients (12–23).

Unlike other autoimmune disorders, such as type 1 diabetes mellitus and autoimmune thyroid disorders (e.g., Graves’ disease), studies on the immune system and circulating T-cell phenotypes in autoimmune PAI are limited and often controversial (24, 25). Understanding which immune cells, factors, and pathways are involved in the progression of the disease is crucial for identifying new therapeutic strategies. We believe that highlighting potential differences in responses to distinct therapeutic strategies could help identify novel mechanisms underlying disease progression and provide valuable insights for the prevention of PAI.

Based on this evidence, we aimed to investigate whether conventional GC replacement regimens or switching to DR-HC elicits different responses in terms of anti-inflammatory and immunomodulatory effects on peripheral circulating lymphocytes and serum parameters.

The primary outcome of the current study was the evaluation of the difference in CD4^+^/CD8^+^ T-cell frequency and CD4^+^/CD25^+^ Treg population in patients with PAI at baseline and after 12 months of GC replacement treatment (conventional and DR-HC).

Secondary outcomes included the evaluation of Treg promotion in terms of the percentage of the Foxp3^+^ gated subset within CD4^+^/CD25^+^ T cells, as well as the assessment of biochemical inflammation parameters and gene expression levels of IL-6, IL-17A, COX-2, HSP-70, IDO, PD-L1, inducible nitric oxide synthase (iNOS), and thioredoxin (TXN)-1.

## 2 Materials and methods

### 2.1 Patients

We prospectively included 30 patients with a diagnosis of autoimmune PAI, for at least 12 months, who were on conventional GC treatment (8 on cortisone acetate and 22 on hydrocortisone) administered twice a day. Among them, 15 consecutive patients were instructed to maintain conventional treatment (4 on cortisone acetate and 11 on hydrocortisone), while 15 patients were switched to DR-HC. Patients were enrolled from July 2018 to December 2021.

The switch to DR-HC was judged to be appropriate on clinical grounds in those patients who complained of fatigue and weakness, presented hyponatremia (<134 mmol/L) or hypoglycemia (≤2.78 mmol/L), or showed more than two comorbidities such as diabetes, osteoporosis/osteopenia, arterial hypertension, and central obesity. The switch from HC to DR-HC was made with an equivalent dose, while the dose was reduced from cortisone acetate to DR-HC taking into consideration the minor glucocorticoid activity of cortisone acetate compared to HC and patients’ clinical characteristics. The study was carried out in accordance with the recommendations of the Paolo Giaccone Policlinico Ethics Committee. All patients signed the informed consent, in accordance with the Declaration of Helsinki.

The study was approved by the ethics committee (No. 07/2018) in the context of a previous study, and an amendment to the study was made after it was approved (No. 01/2019) (26). Adrenal insufficiency was diagnosed according to international guidelines on the diagnosis of adrenal insufficiency (27).

Antibodies against the steroidogenic enzyme 21-hydroxylase and other autoimmune polyendocrine deficiencies (thyroid disease, premature ovarian failure, type 1 diabetes mellitus, hypoparathyroidism, candidiasis) were evaluated as recommended by international guidelines (28).

Eligible subjects were women and men, aged 18–65 years, who had been diagnosed within at least 12 months (mean duration of disease 2.05 ± 1.57 years) and were on stable glucocorticoid replacement therapy. All patients were on fludrocortisone treatment at a mean dose of 0.1 mg/day. Renin levels were routinely used as a marker to guide potential adjustments in fludrocortisone therapy (29). Patients with adrenocortical carcinoma and secondary adrenal insufficiency, patients with PAI on steroid conventional treatment three times a day, and patients who were pregnant were excluded from the study.

Twelve out of the 30 enrolled patients were women, with isolated PAI (nine patients) or APS (three patients). The three patients with APS included one patient with combined celiac disease and PAI, carrying AIRE mutations that confirmed the clinical diagnosis for APS-1 (30), and two patients with combined type 1 diabetes mellitus, hypothyroidism, and PAI. Among the patients with APS, one had combined PAI, type 1 diabetes mellitus, and hypothyroidism and five had combined hypothyroidism and PAI.

Patients were instructed to double the dose of conventional GCs or DR-HC during intercurrent illness or stress (depending on the severity of symptoms) or to practice IM or IV therapy in case of major surgical stress or gastrointestinal disease.

An age-matched group of 20 healthy volunteers (HD) (1:1.5 ratio to patients with PAI; 13 women and 7 men; mean age 32.4 ± 10.5 years) who were adrenal sufficient was recruited at baseline. HD were volunteers without a personal or family history of autoimmune diseases and were randomly recruited.

During the 12 months of the observation period, replacement therapy was stable and was only rarely modified based on clinical judgment of the needs of patients in both groups, with the smallest dose sufficient to provide adequate clinical wellbeing. The mean dose of steroid replacement therapy at 12 months was not statistically significant from the baseline dose.

### 2.2 Study design

Patients were instructed to take the first dose of treatment on waking and the last dose of conventional GC not later than 6 p.m. Blood samples were collected in the morning after overnight fasting and at least 2 h after intake. During the study, we monitored any adverse effects and adrenal crises or hospitalization.

Twenty healthy controls were evaluated only at the time of enrolment. Anthropometric (weight, BMI, and waist circumference), serum metabolic (glucose and lipid levels), and inflammatory parameters (C-reactive protein, lymphocyte-to-neutrophil ratio, fibrinogen, and erythrocyte sedimentation rate) were assessed both in the patients and controls.

### 2.3 Isolation of peripheral blood mononuclear cells

Peripheral blood mononuclear cells (PBMCs) were freshly isolated from the whole blood of 15 subjects on conventional replacement steroids (CRS), 15 subjects on DR-HC, and 20 healthy controls by Ficoll-Paque density gradient centrifugation at two different time points, baseline (T0) and 12 months (_T1_).

### 2.4 Isolation of total RNA and qRT-PCR

PBMC total RNA was extracted and purified using the RNeasy Micro Kit (Qiagen, Milan, Italy), according to the manufacturer’s protocol. For quantitative and qualitative analyses, a NanoDrop 2000 spectrophotometer (Thermo Fisher Scientific, Rodano (MI), Italy) was used: the purity was estimated by 260/280 nm of absorbance and an amount of 2 µg total RNA was reverse-transcribed in a total volume of 20 µL with Oligo dT primers (Promega Italia s.r.l., Milan, Italy) and ImProm-II™ Reverse Transcription System (Promega Italia s.r.l., Milan, Italy). The primer sequences and details are listed in **Table 1**. All reactions were performed using the QuantiTect SYBR Green PCR Kit (Qiagen, California, USA) on the Rotor-Gene Q Instrument (Qiagen, California, USA). Briefly, the amplification conditions were as follows: 95°C for 3 min, 95°C for 20 s, 60°C for 30 s, and 72°C for 60 s. Each reaction was performed at least in triplicate. The specificity of the amplified products was determined by melting peak analysis. Relative mRNA expression for each gene was analyzed using the ^ΔΔ^Ct method according to the guidelines of Livak and Schmittgen (31). All reactions were performed at least in triplicate.

**Table 1:** The primer sequence list used for mRNA expression analysis.

Gene	Product name/QuantiTect primer	GeneGlobe ID
COX-2	Hs_PTGS2_1	QT00040586
HSP-70	Hs_HSPA1A_1	QT01002568
IL-6	Hs_IL6_1	QT00083720
IL-17A	Hs_IL17A_1	QT00009233
IDO	Hs_IDO1_1	QT00000504
PD-L1	Hs_CD274_1	QT00082775
iNOS	Hs_NOS1_1	QT00043372
Thioredoxin-1	Hs_TXN_1C	QT00059332

### 2.5 Protein interaction networks

Network analysis was performed using the STRING (Search Tool for the Retrieval of Interacting Genes/Proteins) website (http://string-db.org/). The co-mentions, co-expression, and associations in curated databases were set as evidence for functional links.

### 2.6 Flow cytometry: T-cell phenotyping

Freshly isolated PBMCs were treated with an FcR blocking reagent (Miltenyi Biotec, Germany) and incubated with each primary fluorochrome-conjugated antibody or appropriate isotype control at 4°C for 30 min in the dark. Intracellular staining for FoxP3 PE was performed using BD Cytofix/Cytoperm Plus Fixation/Permeabilization Kit (BD Biosciences, Milan, Italy) according to the manufacturer’s instructions. The Treg phenotype was determined as a positive stain for CD25PerCP-Cy™5.5, CD4 FITC, and FoxP3 PE by flow cytometry. All data were acquired on a FACSAria™ III Cell Sorter and analyzed using FACSDiva™ 7 software (BD Pharmingen, San Jose, CA Italy). For the gating strategy, lymphocyte populations were gated based on light scattering characteristics using side scatter (SSC) and forward scatter (FSC), and the total lymphocyte population was defined by CD4 expression. Within the lymphocyte cell population P1, CD4^+^/CD25^+^ positive T cells were assessed (Q2). Within Q2, positive cells for Foxp3, defined as Tregs, were distinguished using SSC versus the fluorescence of the relevant fluorochrome (PE). The Tregs were defined as CD25^+^ and Foxp3^+^ in CD4^+^ T cells. The primary antibodies and their related isotype controls were reported in **Table 2**.

**Table 2:** Primary antibodies list used for cytofluorometric analysis.

Primary antibody	Product	Code
CD8	Clone RPA-T8 PE	555367, BD Biosciences
CD25	Clone M-A251 PerCP-Cy™5.5	560503, BD Biosciences
CD4	Clone SK3 BD™ CD4 FITC	566911, BD Biosciences
FoxP3	Clone 259D/C7 PE	560046, BD Biosciences
Isotype control PerCP-Cy5.5	Mouse IgG1	550795, BD Biosciences
Isotype control FITC	Mouse IgG1	555748, BD Biosciences
Isotype control PE	Mouse IgG1	560046, BD Biosciences

### 2.7 Assays

Glucose, lipids, blood count, erythrocyte sedimentation rate, C-reactive protein, and fibrinogen were measured with standard methods (Modular P800, Roche, Milan).

### 2.8 Statistical analysis

The Statistical Package for Social Science (SPSS) version 19 (SPSS, Inc., IBM, New York, USA) was used for data analysis. The normality of quantitative variables was tested with the Shapiro–Wilk test. The baseline characteristics of the groups were presented as mean ± SD for continuous variables, while the rates and proportions were calculated for categorical data. The differences between 12 months and baseline were evaluated by ANOVA analysis. Flow cytometric and qRT-PCR data were expressed as median and interquartile range determined using a non-parametric analysis by GraphPad Software Inc., California. A *p*-value <0.05 was considered statistically significant.

## 3 Results

### 3.1 T-cell phenotyping and regulatory T-cell subpopulation in PAI patients at baseline and after 12 months of conventional replacement glucocorticoid therapy or DR-HC

PBMCs from 30 patients with PAI and 20 HD were analyzed by flow cytometry to investigate potential differences in immunophenotypic patterns. Specifically, we performed a comparative analysis to examine CD4^+^ and CD8^+^ populations and the CD25^+^ subset gated within the CD4^+^ population at baseline in freshly isolated PBMCs from the PAI and HD groups.

**Figure 1:**
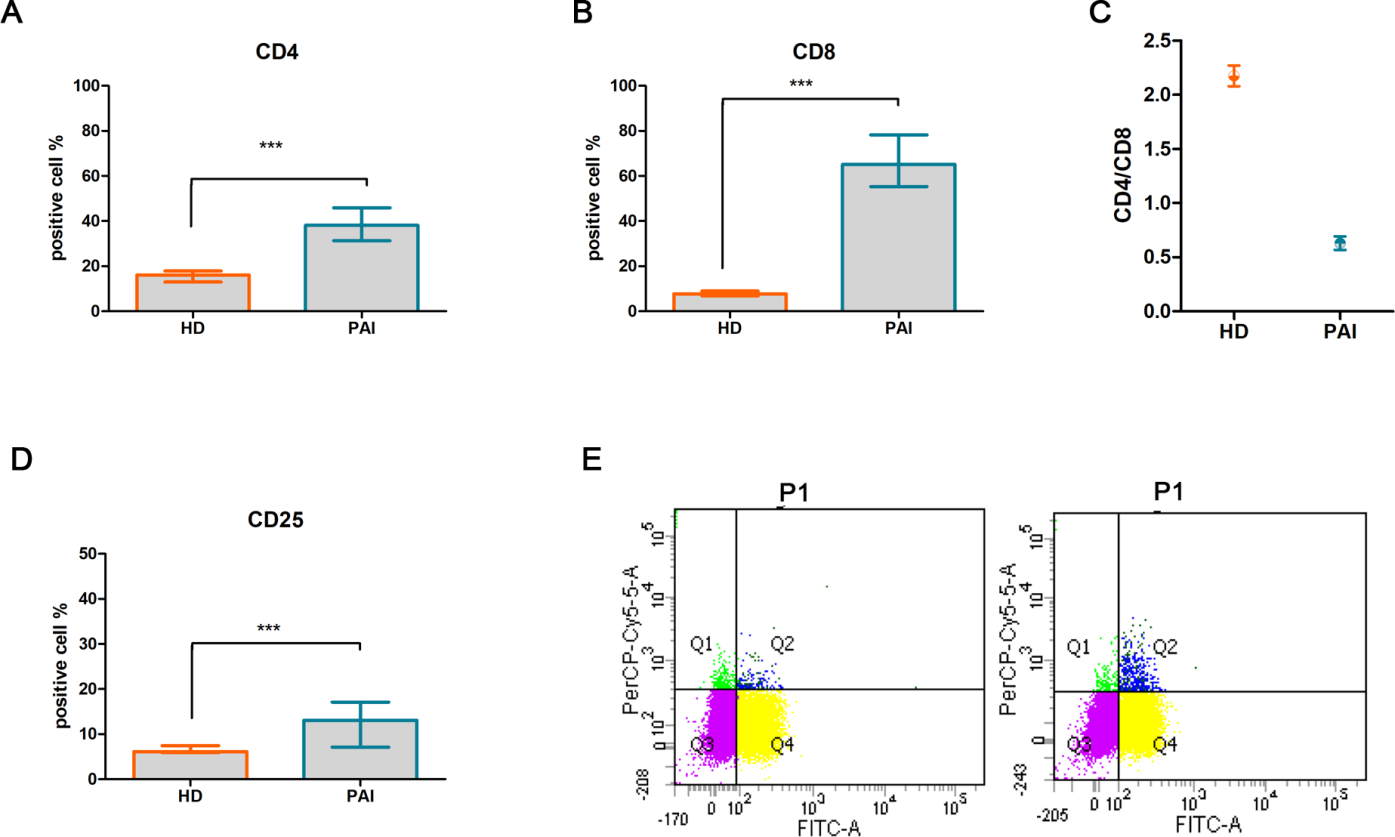
Comparative analysis of activation T-cell markers CD4^+^ **(A)** and CD8^+^ **(B)**, CD4^+^/CD8^+^ ratio **(C)**, T-cell activation marker CD25^+^ **(D)**, and double-positive CD4^+^/CD25^+^ T subpopulation in PBMCs isolated from healthy donors (HD) and patients suffering from PAI disease (PAI) at baseline **(E)**. The histogram (mean and SD Max) and dot plot graphs (mean and SD Max and Min to Max) were performed by Prism v5 (GraphPad Software, Inc, California) and are represented as mean ± SD; *p* = *p*-value, *** ≤0.001, n.s, not significant (*p* > 0.05).

A significant difference was observed in the percentage of CD4^+^ [median (Mdn): 38.14%, interquartile range (IQR): 4.86, *p* < 0.0001, **Figure 1A**] and CD8^+^ (Mdn: 65.13% and IQR: 7.74 vs. Mdn 7. 7 and IQR: 2.1, *p* < 0.0001, **Figure 1B**) in patients with PAI compared to the HD group. An inversion of the CD4^+^/CD8^+^ ratio was found in PAI compared to the HD group (0.63 ± 0.06 vs. 1.99 ± 0.37, *p* < 0.05, **Figure 1C**). Flow cytometry analysis also revealed a higher percentage of CD25^+^ cells (13.08%, IQR: 3.39 vs. 6.1%, IQR: 1.5, *p* < 0.0001, **Figure 1D**), with a representation of 12.92% ± 2.76% vs. 6.09% ± 1.41% of double-positive CD4^+^/CD25^+^ T cells (**Figure 1E**) in PAI compared to the HD group.

**Figure 2:**
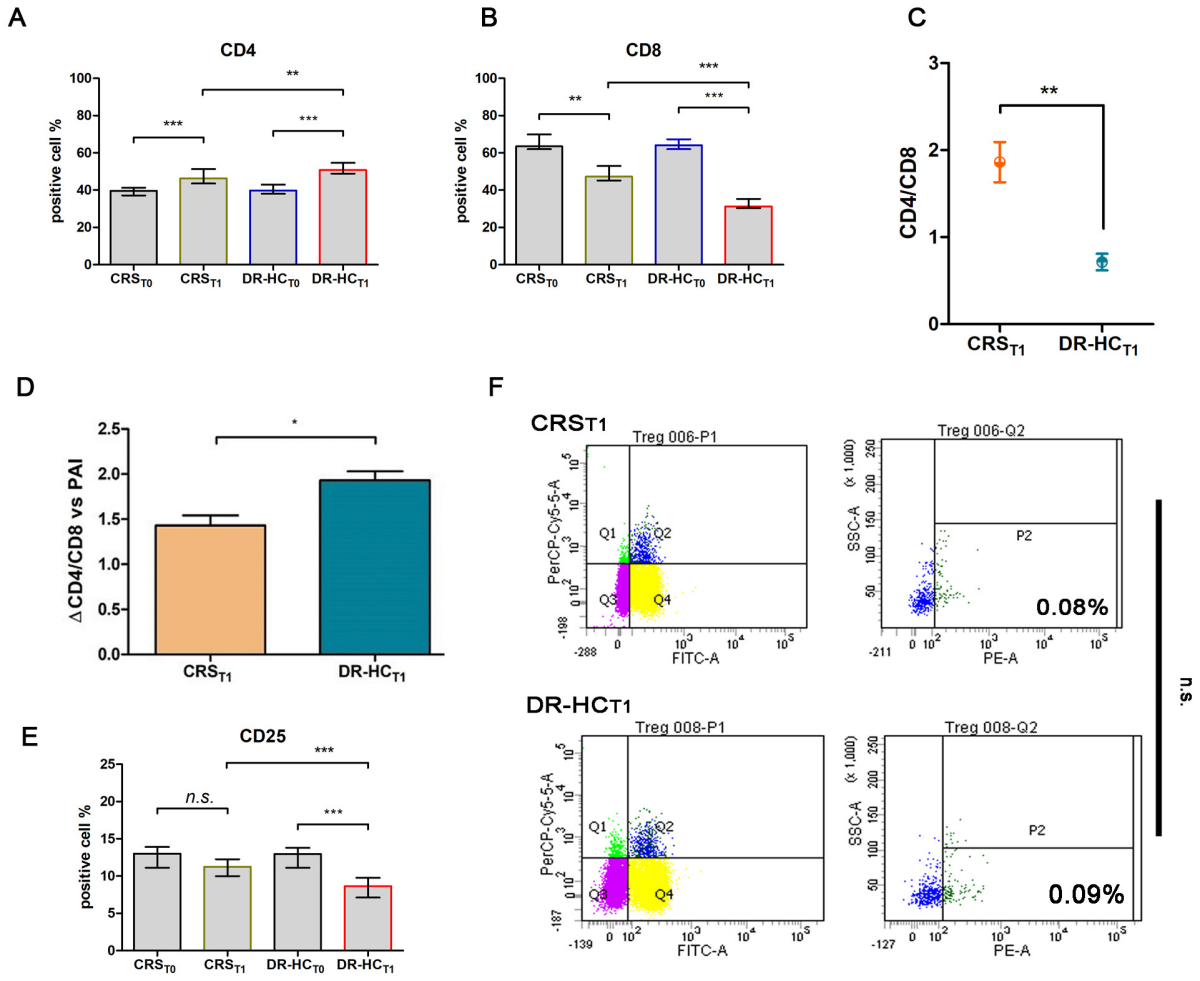
Comparative analysis of activation T-cell markers CD4^+^ **(A)** and CD8^+^ **(B)** in the dual-release hydrocortisone (DR-HC) group compared to the conventional replacement steroid (CRS) group of patients suffering from PAI disease at baseline (T0) and after 12 months of therapy (_T1_); comparative analysis of CD4^+^/CD8^+^ ratio **(C)** and ΔCD4^+^/CD8^+^ ratio in the dual-release hydrocortisone (DR-HC) group compared to the conventional replacement steroid (CRS) group of patients suffering from PAI disease **(D)**. Comparative analysis of the Treg marker CD25 in the dual-release hydrocortisone (DR-HC) group compared to the conventional replacement steroid (CRS) group of patients suffering from PAI disease at baseline (T0) and after 12 months of therapy (_T1_) **(E)**. DR-HC Foxp3-positive Treg population in the conventional replacement steroid (CRS) (upper panel) and dual-release hydrocortisone (DR-HC, bottom panel) groups of patients suffering from PAI disease **(F)**. Within the T-cell population P1, CD4^+^/CD25^+^ positive T cells were assessed (Q2). Within Q2, cells positive for Foxp3, defined as Tregs, were distinguished using SSC versus the fluorescence of the relevant fluorochrome (PE). The histogram (mean and SD Max) and dot plot graphs (mean and SD Max and Min to Max) were performed by Prism v5 (GraphPad Software, Inc, California) and are represented as mean ± SD; *p* = *p*-value, * ≤0.05, ** ≤0.01, *** ≤0.001, n.s, not significant (*p* > 0.05).

After 12 months (_T1_) of treatment with dual-release hydrocortisone (DR-HC) or conventional replacement steroid (CRS), comparative flow cytometry analysis of freshly isolated PBMCs revealed a significant increase in CD4^+^ T cells (Mdn: 50.75% and IQR: 9.66 vs. Mdn: 39.75% and IQR: 4.94, *p* = 0.0015, **Figure 2A**), accompanied by a significant decrease in CD8^+^ T cells (Mdn: 31.30% and IQR: 5.03 vs. Mdn: 64.11 and IQR: 5.32, *p* < 0.0001, **Figure 2B**) and a reversion of CD4^+^/CD8^+^ ratio (0.63 ± 0.06 vs. 2.16 ± 0.09, *p* < 0.0001, **Figure 2C**) in PAI compared to baseline.

Notably, in the DR-HC_T1_ group, a greater reversion in the CD4^+^/CD8^+^ ratio was detected compared to the CRS_T1_ group (0.71 ± 0.09 vs. 1.86 ± 0.23, *p* = 0.013, **Figure 2D**). To investigate Treg involvement, the primary full-functional phenotype CD4^+^/CD25^+^/Foxp3^+^ was examined.

Despite a significant decrease in the percentage of CD25^+^ expression in the DR-HC_T1_ group compared to baseline and to CRS_T1_ (Mdn: 8.64, IQR: 2.63 vs. Mdn: 12.95, IQR: 2.68%, *p* = 0.0002 and vs. Mdn: 11.24, IQR: 2.27%, **Figure 2E**), the CD4^+^/CD25^+^/Foxp3^+^ subpopulation did not show significant differences between the DR-HC_T1_ and CRS_T1_ groups (**Figure 2F**).

### 3.2 Expression profile of inflammatory and immune system regulator genes at baseline and after 12 months of conventional replacement glucocorticoid therapy or DR-HC

**Figure 3:**
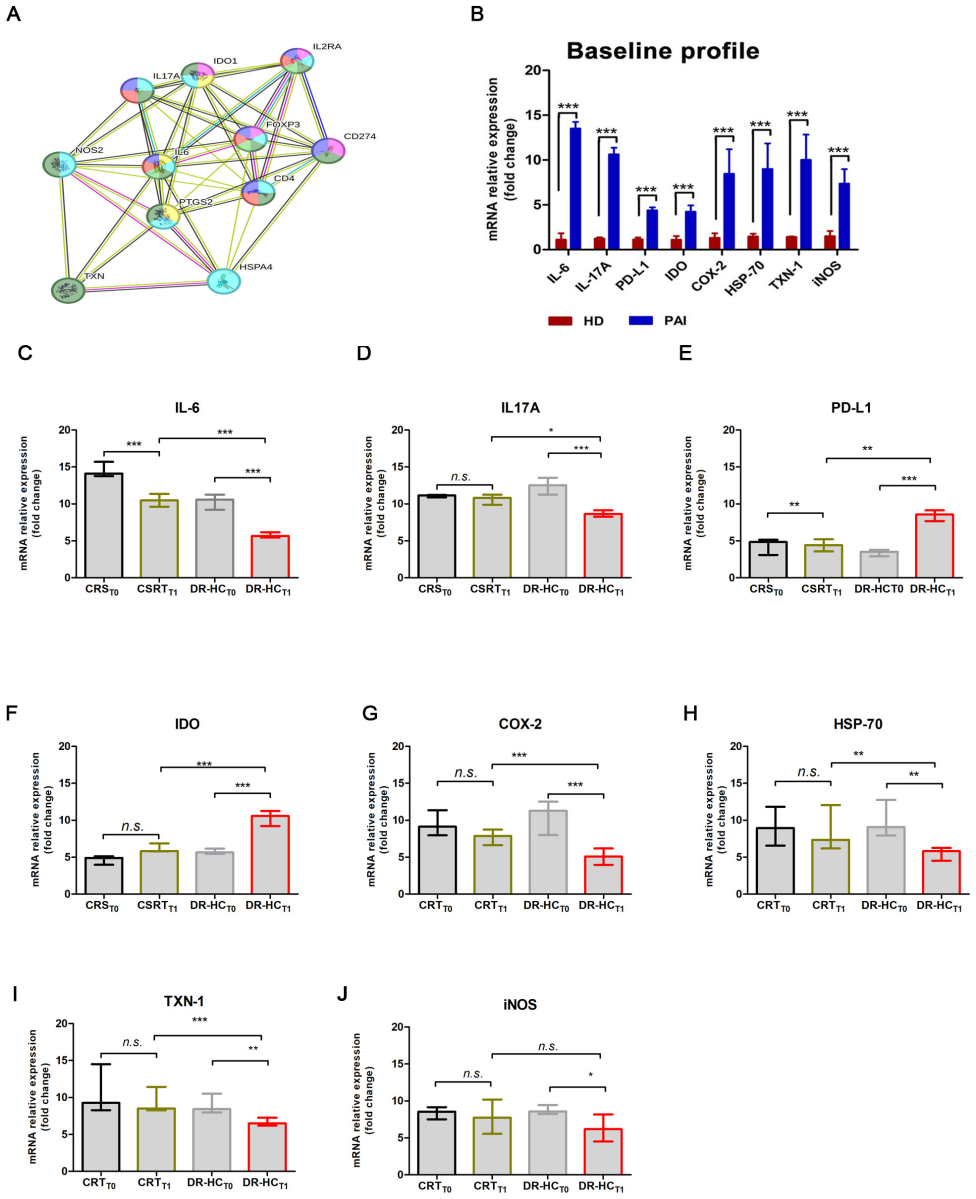
Comparison of mRNA expression levels of inflammatory and immunomodulatory genes in PBMCs from patients suffering from PAI disease after 12 months of conventional replacement steroid (CRS_T1_) or DR-HC (DR-HC_T1_). **(A)** Protein–protein interaction representation by STRING analysis. **(B)** Comparison of mRNA expression levels of inflammatory and immunomodulatory genes in PBMCs from healthy donors (HD) and from patients suffering from PAI disease (PAI) at baseline. Comparative expression level of cytokines IL-6 **(C)** and IL-17A **(D)**, PD-L1 **(E)**, IDO **(F)**, COX-2 **(G)**, HSP-70 **(H)**, TXN-1 **(I)**, and iNOS **(J)** in PBMCs from patients suffering from PAI disease at baseline and after 12 months of conventional replacement steroid (CRS_T1_) or DR-HC (DR-HC_T1_) therapy. The mRNA levels were normalized for the gene β‐actin and expressed as fold change by the ^ΔΔ^Ct method using the Rotor-Gene Q. The histogram graphs (median and interquartile) were performed by Prism v5 (GraphPad Software, Inc, California) and are represented as mean ± SD; *p* = *p*-value, ** ≤0.01, *** ≤0.001, n.s, not significant (*p* > 0.05).

A computational STRING analysis was conducted to investigate the functional interaction between “autoimmunity and inflammation” AND “autoimmune endocrine diseases and T-cell differentiation”. The protein–protein interaction (PPI) network of upregulated immunomodulatory factors and downregulated inflammatory factors identified 56 edges (PPI enrichment *p*-value = 7.9e−15, **Figure 3A**) and 14 nodes. Functional enrichments within the network primarily involved the following processes: “positive regulation of inflammatory response” (*p* = 0.013, yellow), “regulation of inflammatory response” (*p* = 7.7e−05, dark green), “regulation of T-cell activation” (*p* = 7.79e−05, blue), inflammatory response (*p* = 4.33e−05, gray), “regulation of response to stress” (*p* = 0.0003, dark gray), “response to stress” (*p* = 0.0016, pink), “Th17 cell differentiation” (*p* = 2.40e−06, dark green), “sphingolipid biosynthetic process” (*p* = 0.0072, dark pink), and “sphingolipid metabolism” (*p* = 0.014, violet).

Among the primarily involved proteins, IL-6, IL-17A, PD-L1, IDO, COX-2, HSP-70, TXN-1, and iNOS were analyzed by real-time PCR at baseline and after 12 months of therapy, in the CRS and DR-HC groups. At baseline, we observed significantly higher mRNA expression levels of IL-6 (13.52 ± 0.73-fold, *p* < 0.0001), IL-17A (10.64 ± 0.734-fold, *p* < 0.0001), PD-L1 (4.39 ± 0.33-fold, *p* < 0.0001), IDO (4.24 ± 0.7-fold, *p* < 0.0001), COX-2 (8.47 ± 2.73-fold, *p* < 0.0001), HSP-70 (9.001 ± 2.84-fold, *p* < 0.0001), TXN-1 (10.03 ± 2.81-fold, *p* < 0.0001), and iNOS (7.36 ± 1.62-fold, *p* < 0.0001) in PAI patients compared to HD (**Figure 3B**).

After 12 months of treatment, we investigated the immune and anti-inflammatory profiles. A significant decrease in IL-6 (*p* = 0.0028, **Figure 3C**) and IL-17A (*p* = 0.0147, **Figure 3D**), along with a significant increase in PD-L1 (*p* = 0.003, **Figure 3E**), IDO (*p* = 0.0038, **Figure 3F**), COX-2 (*p* < 0.0001, **Figure 3G**), HSP-70 (*p* = 0.0052, **Figure 3H**), and TXN-1 (*p* = 0.0008, **Figure 3I**), was detected. Conversely, iNOS-mRNA levels remain unchanged when comparing the DR-HC_T1_ group to CRS_T1_ (**Figure 3J**).

Additionally, we observed a significant downregulation of IL-6 by approximately 0.46-fold in the CRS_T1_ group and 1.16-fold in the DR-HC_T1_ group (*p* = 0.037 and *p* = 0.0019, respectively, **Figure 3C**). Meanwhile, IL-17A mRNA levels were significantly downregulated only in the DR-HC_T1_ group compared to baseline (≈0.29-fold, *p* = 0.046, **Figure 3D**). Two major immunomodulatory factors PD-L1 and IDO were upregulated by approximately 1.25-fold (*p* < 0.0001, **Figure 3E**) and 1.09-fold (*p* < 0.0001, **Figure 3F**), respectively. In contrast, a significant downmodulation of COX-2, HSP-70, and TXN-1 by approximately 0.56-fold (*p* < 0.0001), 0.27-fold (*p* = 0.006), and 0.18-fold (*p* = 0.008) (**Figures 3G–I**) was observed in the DR-HC_T1_ group compared to baseline.

### 3.3 Clinical and biochemical analyses

Baseline characteristics were similar for patients with PAI and controls (**Table 3**).

**Table 3:** General characteristics of all patients with adrenal insufficiency and in patients treated with conventional treatment and dual-release hydrocortisone treatment at baseline.

	Patients with adrenal insufficiency (*N* = 30)	Conventional treatment (*N* = 15)	DR-HC treatment (*N* = 15)	p*
	Subjects (%)	Subjects (%)	Subjects (%)	
Gender Male	18 (60%)	8 (53.3%)	10 (66.7%)	0.723
Female	12 (40%)	7 (46.7%)	5 (33.3%)	
Isolated primary adrenal insufficiency	21 (70%)	12 (80%)	9 (60%)	0.239
Thyroid autoimmune disease	3 (10%)	2 (13.3%)	1 (6.6%)	0.546
Celiac disease	8 (26.6%)	5 (33.3%)	3 (20%)	0.418
Type 1 diabetes mellitus	1 (3.3%)	1 (6.6%)	0	0.331
	Mean ± SD	Mean ± SD	Mean ± SD	
Mean equivalent dose of hydrocortisone (mg/day)	22.4 ± 5.77	23.4 ± 5.42	22.7 ± 6.21	0.258
Age (years)	40.3 ± 19.3	43.3 ± 18.9	37.8 ± 19.5	0.203
BMI (kg/m^2^)	25.4 ± 5.86	24.3 ± 3.47	24.1 ± 4.17	0.363
Waist circumference (cm)	95.8 ± 13.9	95.9 ± 12.3	96.5 ± 10.9	0.696
Na (mmol/L)	139.2 ± 4.8	139.6 ± 4.1	138.9 ± 5.41	0.573
K (mmol/L)	4.6 ± 0.53	4.67 ± 0.59	4.54 ± 0.42	0.628
Total cholesterol (mmol/L)	5.2 ± 1.21	4.83 ± 1.1	5.21 ± 1.21	0.165
HDL cholesterol (mmol/L)	1.41 ± 0.47	1.35 ± 0.49	1.38 ± 0.48	0.403
Triglycerides (mmol/L)	1.68 ± 0.94	1.43 ± 0.88	1.46 ± 0.99	0.476
LDL cholesterol (mmol/L)	3.04 ± 1.13	2.71 ± 1.11	2.78 ± 1.01	0.114
HbA1c (mmol/moL)	42.1 ± 0.8	42 ± 0.85	41 ± 0.75	0.685
Fasting glycemia (mmol/L)	4.95 ± 2.03	4.96 ± 0.79	5.07 ± 0.95	0.277
C-reactive protein (mg/L)	4.23 ± 2.75	4.45 ± 2.25	4.02 ± 3.25	0.121
Erythrocyte sedimentation rate (mm)	12.3 ± 8.72	11.9 ± 9.87	12.8 ± 7.59	0.791
Fibrinogen (mg/dL)	315.6 ± 87.6	260 ± 156.7	359.2 ± 76.6	0.179
Neutrophil/lymphocyte ratio	1.49 ± 0.7	1.41 ± 0.71	1.56 ± 0.69	0.104

In patients treated with DR-HC, a significant decrease in waist circumference (WC) (*p* = 0.048) was observed at 12 months compared to baseline (**Table 4**). We also showed significantly lower WC (*p* = 0.029), glucose (*p* = 0.042), C-reactive protein (*p* = 0.024), erythrocyte sedimentation rate (*p* = 0.022), and neutrophile-to-lymphocyte ratio (*p* = 0.032) in patients treated with DR-HC compared to conventional treatment (**Table 4**). We also investigated a potential correlation between WC and CD4^+^ (*p* = 0.147; *r* = 0.784) and CD8^+^ T cells (*p* = 0.185; *r* = 0.803), without significant findings.

**Table 4:** Anthropometric, metabolic, and inflammatory parameters in patients on conventional steroid replacement treatment and dual-release hydrocortisone (DR-HC) at baseline and after 12 months of treatment.

	Conventional steroid treatment (*N* = 15)			Dual-release hydrocortisone (*N* = 15)			
	Baseline Mean ± SD	12 months Mean ± SD	p*	Baseline Mean ± SD	12 months Mean ± SD	p**	p***
Mean equivalent dose of hydrocortisone (mg/day)	23.4 ± 5.42	24.1 ± 5.28	0.722	22.7 ± 6.21	23.5 ± 5.82	0.718	0.769
Anthropometric parameters							
BMI (kg/m^2^)	24.3 ± 3.47	25.4 ± 4.18	0.439	24.1 ± 4.17	22.4 ± 4.06	0.267	0.056
WC (cm)	95.9 ± 12.3	98.9 ± 12.8	0.483	96.5 ± 10.9	89.1 ± 10.5	0.048	0.029
Total cholesterol (mmol/L)	4.83 ± 1.1	4.98 ± 1.26	0.741	5.21 ± 1.21	4.82 ± 1.15	0.377	0.723
HDL cholesterol (mmol/L)	1.35 ± 0.49	1.37 ± 0.48	0.872	1.38 ± 0.48	1.49 ± 0.47	0.516	0.488
Triglycerides (mmol/L)	1.43 ± 0.88	1.66 ± 0.93	0.484	1.46 ± 0.99	1.55 ± 0.66	0.767	0.722
LDL cholesterol (mmol/L)	2.71 ± 1.11	2.88 ± 1.03	0.671	2.78 ± 1	2.51 ± 1.14	0.501	0.365
HbA1c (mmol/moL)	42 ± 0.8	43 ± 0.7	0.853	41 ± 0.75	38 ± 0.7	0.524	0.292
Fasting glycemia (mmol/L)	4.96 ± 0.79	5.47 ± 0.81	0.062	5.07 ± 0.95	4.58 ± 0.75	0.124	0.042
Inflammatory markers							
C-reactive protein (mg/L)	4.45 ± 2.25	7.41 ± 3.85	0.166	4.02 ± 3.25	4.5 ± 2.75	0.665	0.024
Erythrocyte sedimentation rate (mm)	11.9 ± 9.87	14.5 ± 6.43	0.399	12.8 ± 7.59	8.92 ± 6.23	0.137	0.022
Fibrinogen (mg/dL)	260 ± 156.7	312 ± 198.6	0.432	359.2 ± 76.6	318.7 ± 80.3	0.168	0.934
Neutrophil/lymphocyte ratio	1.41 ± 0.71	1.84 ± 0.79	0.128	1.56 ± 0.69	1.25 ± 0.72	0.238	0.032

*p: changes from baseline to 12 months in the conventional steroid treatment group.

**p: changes from baseline to 12 months in the dual-release hydrocortisone group.

***p: comparison between conventional steroid treatment and dual-release hydrocortisone at 12 months of follow-up.

## 4 Discussion

In humans, data regarding the modulation of the immune response and the inflammatory status in PAI patients compared to healthy controls are limited. In our study, we first analyzed the circulating T lymphocyte subsets in patients with autoimmune PAI and healthy controls and subsequently the changes during different replacement regimens.

We found a higher percentage of CD4^+^ and CD8^+^ T cells and a lower CD4^+^/CD8^+^ ratio in patients with PAI compared to healthy controls, confirming that these cells play a crucial role in the evolution of adrenalitis. Consistent with our findings, Nowotny et al., who evaluated the immune cell subsets in patients with PAI of different etiologies, showed that in AD, T helper, cytotoxic, and natural killer cell subsets were higher compared to controls (32).

Characterizing the different T-cell subsets is crucial for elucidating the pathogenetic process of PAI. It is well documented that Tregs are involved in the maintenance of self-tolerance, and when they are deficient or dysfunctional, this can favor the development of autoimmune disorders. According to several studies, the development and persistence of autoimmune diseases, such as type 1 diabetes mellitus and rheumatoid arthritis, are associated with a decrease in the Treg population or a defect in the suppressive activity of Treg cells. An increase in the number of fully active Treg cells may be beneficial in autoimmune disorders (33).

In autoimmune PAI, there is a gradual destruction of adrenal glands, resulting in poor and inadequate hormone production (34, 35). In patients with AIRE mutations, defective and reduced Tregs have been observed, while conflicting data are available for patients with isolated PAI (36).

In the present study, we compared the T-cell profile between patients with PAI after 12 months of conventional GC replacement therapy and DR-HC treatment. Following 12 months of DR-HC therapy, we observed a decrease in the number of CD8^+^ T cells and a normalization of the CD4^+^/CD8^+^ ratio to greater than 1, aligning more closely with a physiological condition and indicative of improved immune function. Although there was a significant increase in the CD4^+^ T-cell population, this was not associated with a promotion of the CD4^+^/CD25^+^ subpopulation. Additionally, we identified a higher CD4^+^/CD25^+^ Treg subpopulation in patients with PAI compared to controls; however, no significant changes were observed in the CD4^+^/CD25^+^/Foxp3^+^ Treg population between CSR and DR-HC after 12 months.

These findings are consistent with those of Sjogren et al., who did not observe differences in the number and metabolic capacities of Tregs in patients with PAI compared to healthy controls, suggesting that functional impairments may contribute to the autoimmune setting (37). Several studies have also investigated the effects of GC replacement therapy on the immune system in patients with adrenal insufficiency, although the results are quite controversial, likely due to varying study methodologies and the different parameters assessed. Bancos et al. demonstrated that patients with adrenal insufficiency receiving a hydrocortisone replacement regimen three times daily experienced a selective impairment of natural killer cell cytotoxicity (38). Isidori et al. studied the changes in the immune system in patients with adrenal insufficiency after switching from multiple daily doses to a once-daily hydrocortisone regimen. In this single-blind randomized controlled trial, patients on conventional replacement therapy exhibited an increased number of classical monocytes (CD14^+^CD16^−^) and a reduced number of CD16^+^ NK cells. This immune pattern was reversed after switching from the multidose regimen to the single-dose hydrocortisone regimen, likely correlated to a resynchronization of circadian rhythm and clock genes (39). The frequency of infections also improved in patients who switched to once-daily dosing, suggesting that the most harmful metabolic and cardiovascular effects of glucocorticoid replacement therapy are associated with a low-grade inflammatory state, which can be reversed by modifying the timing of administration (20). However, data regarding the immune and inflammatory response adaptation to different therapies remain missing.

The modulation of the immune response is primarily mediated by the change in the expression of several modulator factors, which are also released by T cells, including cytokines and inflammatory proteins. Among these, PD-L1 interacts with its receptor PD-1 to inhibit Th1 lymphocyte proliferation and promote their conversion into Treg cells (4, 5). PD-L1 is also expressed on human PBMCs, and its expression has been identified in several autoimmune disorders, functionally protecting against autoimmune disorders and preventing disease progression (40). Indeed, the use of anti-PD-1/PD-L1 inhibitors, as pharmacological agents, has been reported to cause adrenal insufficiency (41).

Similar to PD-L1, IDO released by PBMCs is involved in immunological tolerance, including the regulation of Treg function (6). IDO is a signaling protein that reflects the immunological microenvironment *in vivo*, playing a crucial role in inhibiting Th17-like effector cells while simultaneously inhibiting IL-6 secretion and reducing infiltrating CD8^+^ T cells (7).

Once the self-tolerance collapses, autoreactive Th1 lymphocytes release several cytokines, including IL-6 and IL-17A, which contribute to the inflammatory state of the microenvironment, altering the balance of COX-2, HSP-70, iNOS, and TXN-1 (6, 8, 26). HSP-70 is an inducible protein whose expression is upregulated under conditions of cellular stress, involved in cell repair, survival, and the prevention of protein aggregation. Under cellular stress, HSP-70 rapidly accumulates first in the nucleus and then in the cytoplasm of cells. Extracellular HSP-70 exhibits proinflammatory activity when acting as an element of immune signaling pathways (8). COX-2 is an inducible enzyme whose upregulation is associated with a proinflammatory immune response, generating and maintaining inflammatory reactions through the production of prostaglandins, prostacyclin, and thromboxane (42, 43). TXN-1 was recently described as a novel key player in the expansion of T cells (9).

Our data revealed that, at baseline, patients with PAI on conventional GC replacement therapy exhibited an increased proinflammatory status, with elevated levels of inflammatory markers (IL-6, IL-17A, COX-2, HSP-70, TNX-1) and immunomodulatory parameters (PD-L1, IDO, and INOS), compared to healthy controls. After switching to DR-HC treatment, a significant improvement in the immune and inflammatory response was observed. Interestingly, 12 months of DR-HC treatment led to a decrease in the expression of proinflammatory IL-6 and IL-17A (which are able to induce the differentiation of CD4^+^ T cells to Th17 cells and reduce Treg cell differentiation) (44) and was associated with a significant downregulation of COX-2, HSP-70, and TNX-1.

Additionally, we demonstrated that DR-HC was associated with an improvement in BMI and a significant decrease in WC. An interconnection between obesity and the immune system has been suggested. Schmitz et al. showed a significant inverse correlation between CD4^+^ Treg populations and some anthropometric parameters including BMI, WC, relative visceral adipose tissue, and waist-to-hip ratio, suggesting that obesity affects the immune system (45). In the present study, we did not find any correlation between anthropometric parameters and CD4^+^ and CD8^+^ T cells. Few studies have examined the link between obesity, body composition, and Tregs, with inconsistent results. Some found reduced Tregs in obesity and improved insulin sensitivity with Treg induction, while others reported body mass-dependent Treg depletion in adipose tissue (45–48).

Our comprehensive PPI analysis highlighted a potential relationship between inflammatory factors (COX-2, HSP-70, TXN-1) and sphingolipid biosynthesis, including serine palmitoyl transferase long-chain base subunit-1 (SPTLC1) and glucocerebrosidase-1 (GBA). This supports the hypothesis that a different CD4^+^/CD8^+^ T regulation could be established. Beyond their role in structural function, sphingolipids also act as key second messengers in various cell types, including T cells (49). Abimannan et al. suggested a potential role of *de-novo* sphingolipid biosynthesis in altering intracellular oxidative balance, leading to an enhancement of Th17 cell frequency in autoimmune diseases (50). Moreover, the bioactive role of sphingolipids in glucocorticoid metabolism and their involvement in various endocrine disorders have been described (51, 52).

In summary, our results suggest that the improvement in immune response is independent of Treg promotion or may be mediated by a Treg subset with a different phenotype. This could support the hypothesis that DR-HC restores a physiological rhythm of Treg expansion, making it more aligned with normal physiological conditions.

Furthermore, we also observed a significant decrease in serum inflammatory parameters after 12 months of treatment with DR-HC compared to conventional GC therapy, which to our knowledge represents an innovative finding. The regulation of inflammatory indices is crucial for maintaining immune homeostasis. Based on our results, it could be also hypothesized that the restoration of circadian rhythm and the resynchronization of the clock gene, likely induced by DR-HC treatment as suggested by Isidori et al. (20), could contribute to the improvement of the inflammatory response, independent of Treg promotion.

The current study has some limitations. First, we only investigated the subsets of circulating lymphocytes mentioned and did not include other subsets that were studied in other research. Additionally, the panel of cytokines we evaluated was not exhaustive. We focused on IL-6 and IL-17A as the main proinflammatory cytokines, excluding other potential cytokines. Similarly, we evaluated Foxp3^+^, in conjunction with CD4^+^CD25^+^, although these are not the only markers for identifying Tregs. Second, we only investigated two time points (i.e., baseline and 12 months after the switching). Future studies should employ a repeated measures design to provide further insight into the changes in circulating lymphocyte subsets. Another limitation concerns the correlation between adrenal-infiltrating and circulating lymphocytes. A paired analysis of circulating T lymphocytes and corresponding adrenal-infiltrating subtypes should be conducted in future studies to identify the cell subsets that could serve as biomarkers in peripheral blood. Fourth, our results may be at risk of bias due to the non-randomization of the study. Fifth, a small number of patients was included in the study, although adrenal insufficiency and, notably, APS are rare conditions affecting 1 in 5,000–7,000 individuals in the general population. Finally, there is heterogeneity among the patients, as we included both patients with isolated PAI and those with APS, despite a similar disease duration. Among the APS patients, only one had APS-1, a condition that could affect the balance between CD4/CD8 or CD4/CD25 cells.

Sixth, all patients included in the study were treated with a twice-day conventional steroid replacement therapy. We acknowledge that HC has a shorter half-life compared to cortisone acetate, and some patients may require a thrice-daily dosing schedule to adequately mimic the physiological cortisol profile. In our study, we aimed to standardize treatment with twice-daily HC dosing, which is commonly used in clinical practice. However, we recognize that this regimen may not be sufficient for all patients, particularly those with higher metabolic demands or variable absorption rates. Despite these limitations, the strength of the study lies in the fact that the switch from multiple daily doses of immediate-release cortisone or hydrocortisone to DR-HC treatment was able to change T lymphocyte phenotype and CD4^+^/CD8^+^ ratio, inducing a significant improvement in the immune and inflammatory pattern in adult patients with autoimmune PAI, not at the onset of the disease. Specifically, to our knowledge, this is the first time that the panel of inflammatory and immune modulators (TXN-1, HSP-70, and COX-2 and IL-6, IL-17A, PD-L1, and IDO) we have selected has been analyzed in patients with autoimmune PAI.

## 5 Conclusions

Patients with autoimmune PAI exhibit a lower CD4^+^/CD8^+^ ratio compared to healthy controls. Treatment with DR-HC is associated with a normalization of the CD4^+^/CD8^+^ ratio, in contrast to patients treated with conventional GC treatment, who maintain a low CD4^+^/CD8^+^ ratio.

The use of DR-HC promotes an increase in PD-L1 expression and a decrease in TXN-1, which inhibits lymphocyte proliferation, as well as a reduction in the expression levels of COX-2 and HSP-70. However, this is not accompanied by enhanced production of Th2-associated cytokines. Although a significant increase in IDO was observed, no significant differences were found in the percentage of Foxp3^+^ Tregs in patients treated with DR-HC compared to those on conventional GCs, suggesting that the role of Tregs in the maintenance of adrenal insufficiency remains unclear and warrants further investigation.

The findings of our study suggest that improvement in immune response is independent of Treg promotion, or possibly mediated by a different Treg subset, with a different phenotype. This supports the hypothesis that DR-HC restores a rhythm of Treg expansion, aligning it to a more physiological condition, potentially due to the resynchronization of the circadian cortisol rhythm and clock genes. We also hypothesize that the normalization of the cortisol rhythm halts or prevents the progression of adrenalitis. Finally, our work suggests that the CD4/CD8 ratio might serve as a potential biomarker for immuno-monitoring in adult patients with autoimmune PAI. However, larger studies are needed to confirm our preliminary findings and identify new therapeutic targets.

## Data Availability

The datasets presented in this study can be found in online repositories. The names of the repository/repositories and accession number(s) can be found in the article/supplementary material.
